# Stroke systems of care in the Philippines: Addressing gaps and developing strategies

**DOI:** 10.3389/fneur.2022.1046351

**Published:** 2022-11-24

**Authors:** Maria Epifania Collantes, Jose Navarro, Allan Belen, Robert Gan

**Affiliations:** ^1^Department of Neurosciences, University of the Philippines Manila-Philippine General Hospital, Manila, Philippines; ^2^Department of Behavioral Medicine and Neurosciences, Santo Tomas University Hospital, Manila, Philippines; ^3^Department of Medicine, Fatima University Medical Centre, Antipolo, Philippines

**Keywords:** stroke care, lower middle income country, Philippines, developing countries, stroke

## Abstract

In the Philippines, the mortality from stroke during the last 10 years remains high. This paper aims to describe the gaps in stroke care and the development of stroke systems of care in the Philippines. Gaps in stroke systems of care include low number of neurologist, inadequate CT scan machines, lack of stroke training among health workers, lack of stroke protocols and pathways, poor community stroke awareness, low government insurance coverage with high out of pocket medical expenses, lack of infrastructure for EMS, inadequate acute stroke ready hospitals, stroke units and rehabilitation facilities. Although there are government programs for primary stroke prevention, the strategies are inadequate to address the stroke pandemic. The Stroke Society of the Philippines has worked with the government for nationwide and regional stroke training of health care workers, community stroke awareness, setting up acute stroke ready hospitals and acute stroke units in different areas of the country and adapting stroke protocols and pathways. Stroke registries are now utilized for quality improvement. Thrombolysis rate has improved from 1.4% in 2014–2016 to 11% in 2021 based on RES-Q database. Because of government subsidy, thrombolysis in the government hospitals is higher at 7.4% (range 4.4–16.9) compared to 4.8% (range 0–10.1) rate in private hospitals. Mechanical thrombectomy rate remained low at 0.4% of all acute ischemic stroke patients because of the cost. With limited resources, infrastructures for emergency medical service is lacking. The innovations done by other LMIC can be done in the Philippines including the use of technology to reach out to geographically isolated areas and use of mobile stroke units. Non neurologist can be trained to help treat stroke patients. Upgrading of the Philhealth insurance to cover for reperfusion therapies, adequate stroke infrastructures and network, and increase in community stroke awareness are areas for improvement in the Philippine stroke systems of care.

## Introduction

Eighty six percent (86%) of global stroke death and 89% of stroke related DALYs occur in low and lower-middle income countries (LMIC) (1). Delivery of quality and evidenced-based care for optimal patient outcomes is limited and poor in LMIC compared to high-income countries (2). There is inadequacy of resources and many challenges in setting up stroke services in LMIC. However, the researches on prevention and treatment of stroke have focused primarily on the needs of high-income countries.

The Philippines has a population of 109 million and only 5.7% are above age 65 years old (3). Despite the young population, stroke remains a fatal disease and the second cause of death (3). Even with the advances in science and introduction of government health programs, the mortality remains high. With paucity of good epidemiologic data, the real burden of disease is still not known. While the annual stroke mortality in the country is reported, the number of stroke survivors with the disability has not been evaluated.

Stroke, together with cardiovascular diseases is the biggest pandemic. Many Filipinos suffer from stroke that are preventable and treatable with cost-effective interventions. Despite the increasing stroke prevalence, prevention and treatment including emergency care are inadequately funded and poorly prioritized. The implementation of primary and secondary stroke prevention is difficult without sufficient government funding. There are no standardized acute stroke management. With limited resources, the reperfusion therapies are not accessible to the majority of Filipino stroke victims.

Strategies to improve stroke systems of care require coordinated efforts from the government, the healthcare workers and community. Mortality will remain high without improvement in infrastructures, access to essential medicines, community education and stroke training among health workers. In low resource areas, greater resources should be allocated for stroke prevention.

This paper aims to describe the gaps in stroke care and the development of stroke systems of care in the Philippines.

## Methodology

A search was conducted for recently published studies on stroke epidemiology and stroke care in the Philippines. The search for studies published since January 2000 on stroke in the Philippines was conducted using Medline (2000–September 2022), HERDIN Plus and Google scholar. The search identified any citations containing the words “stroke” or “cerebrovascular” as title words and “Philippines” anywhere in the abstract or citation. The search was limited to English language manuscripts. Abstracts from the searches were screened and papers of stroke epidemiology and care were selected. Additional papers were identified from the reference lists of previously published studies and reviews. The Stroke Society of the Philippines website was accessed for stroke activities in improving stroke awareness and care.

## Results

### Stroke mortality

**Figure 1:**
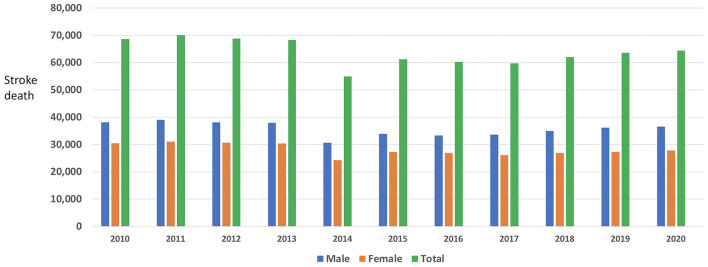
Philippine stroke mortality 2010–2020 (4, 5).

The mortality from stroke during the last 10 years remains high with an average of 63,804 deaths per year (Figure 1) (4). In 2021, despite the COVID pandemic, the recorded annual Philippine stroke death was 68,180 (5), increased from 64,381 in 2020 (4). These data taken from death certificates may not be accurate with under reporting as 34% of Filipinos die without medical attendance (4). About 50% of the population do not have access to primary care facilities within 30 min (6). Uncontrolled risk factors, poor stroke awareness, delay of hospital access, high out-of-pocket medical expenses, overcrowding of public hospitals, inadequate CT scan machines and lack of stroke training among health workers could have contributed to the high stroke mortality rate (7).

### Gaps in stroke system of care in the Philippines

The overall stroke system of care can be assessed at least partly by different factors such as the number of neurologists providing stroke care, the density of imaging facilities in the country, the rate of thrombolysis and the availability of stroke units (8). Furthermore, the stroke continuum of care highlights the importance of stroke rehabilitation in improving outcomes (9). Unfortunately, the current state of the Philippines' stroke system of care is lacking in all of these areas.

In 2021, there is one neurologist providing healthcare needs for every 218,000 Filipinos which is very low compared to the recommended ratio of 106 neurologists per 100,000 population (10). There is also unequal distribution of neurologist with as much as 67% of neurologists are concentrated in the highly urbanized centers in the country (10).

One essential component of stroke diagnosis and management is brain imaging. In the Philippines, the reported density of computed tomography (CT) scan and magnetic resonance imaging (MRI) are 1.09 per million population and 0.30 per million population, respectively (7). There is also a big disparity between the public and private sector as the former has fewer number of machines available for stroke diagnosis (11).

In the Philippines, the use of recombinant tissue plasminogen activator (rTPA) for eligible acute ischemic stroke patients within the 3–4.5 h time window was approved in 1999. However, the utilization of this important drug remains low. In 2016, the department of health (DOH) implemented the Stroke Medicine Access Program (SMAP) which provided more than 1,000 free vials of rTPA to selected government hospitals across the country (12). However, due to gaps in the implementation of SMAP such as lack of healthcare workforce training and facilities for stroke diagnosis and care, the program was put on hold (13). Just recently, alteplase was included in the Philippine National Formulary (PNF) as an essential medicine for stroke care and as such may be reimbursed to the country's national health insurance system, the Philippine Health Insurance Corporation (PhilHealth) (14). Navarro et al. reported a thrombolytic rate of 1.4% from year 2014 to 2016 (15) which is within the lower bracket of 1.3%−9% intravenous rTPA use among other Asian countries (16).

Admitting acute stroke patients in the stroke units (SU) yield better care and improved stroke outcomes (17). Through the efforts of the Stroke Society of the Philippines (SSP), the number of SUs increased from merely two in 1999 to 47 in 2021, However, it is still low compared to the ideal number as recommended by the World Stroke Organization. Majority of SUs are located in urbanized centers and access to care of stroke patients from remote areas is difficult (18).

Rehabilitation after stroke remains as the primary means of which maximal recovery of function may be achieved (9). However, access to stroke rehabilitation services and facilities remains inadequate as there are only 452 rehabilitation centers to serve 148 stroke cases per 100,000 population (19, 20) in the country. Similar to access to neurologists, patients have poor access to post stroke care physiatrists and rehabilitation specialists as they are concentrated in highly urbanized areas. Only 15.8% of hospitals in the Philippines have rehabilitation centers (21). Gonzales-Suarez et al. in their 2015 audit study of hospitalized stroke patients reported that only 54.1% was referred to rehabilitation services despite that the data was collated only in hospitals with rehabilitation centers. This suggests that there is a significant low utilization of stroke rehabilitation services even in centers where it is available. Other than poor access to services and facilities, cost of rehabilitation also poses as a limiting factor. The current PhilHealth packages for stroke cover only medical expenses while rehabilitation costs are borne out of pocket and private health insurance covers only a limited amount of the rehabilitation expenses (22).

### Strategies in improving stroke care

**Figure 2:**
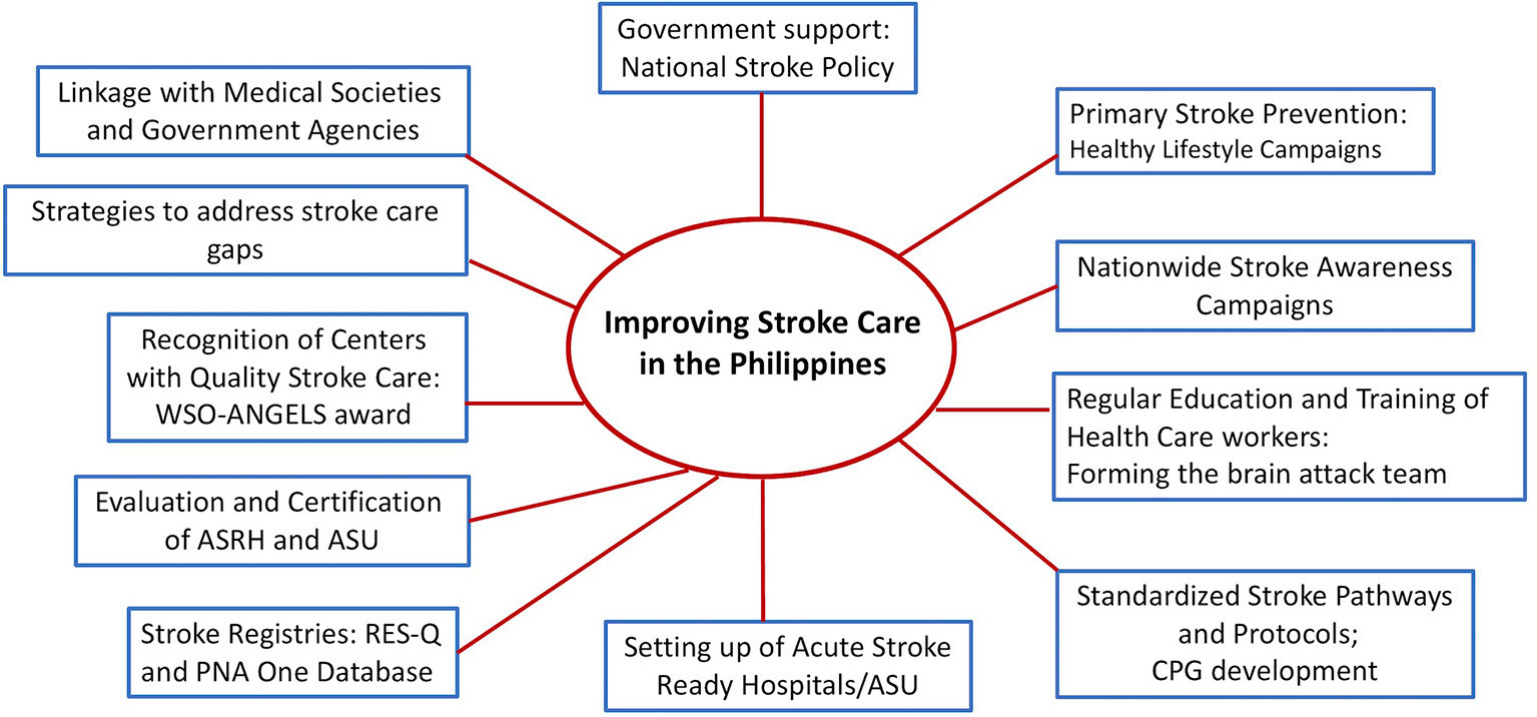
Improving stroke care in the Philippines (20).

There is inequality in stroke care which differs greatly across rural and urban locations. Addressing the stroke care gaps needs government reforms and policies. Figure 2 shows the initial steps done by the government and the Stroke Society of the Philippines (20). There are a lot of challenges and limitations. The impact will be assessed in the next few years.

### National stroke policy

In 2020, the Philippine government through the Department of Health (DOH) approved the Stroke National policy through the creation and implementation of the Administrative Order No. 2020-0059 (23). This mandates the timely and quality stroke care to be delivered to all Filipinos in all health facilities. This will facilitate the standardization of stroke protocol and pathways for diagnosis and treatment. With the national stroke policy, the department of health shall facilitate the referral pathways and utilize health care provider networks to ensure timely referral of patients to ASRH. The government will facilitate capacity building and formation of interdisciplinary teams. Access to essential medicine like rTPA will also be ensured by the government and its attached agencies (23).

### Primary stroke prevention campaigns

In 2011, the DOH implemented the National Policy on Strengthening the Prevention and Control of Chronic Lifestyle Related Non-Communicable Diseases (24). The aim was to adopt a nationwide, integrated and comprehensive program on prevention and control of lifestyle diseases. It targeted the reduction of risk factors like smoking, hypertension and diabetes with a goal of 2% reduction of NCD mortality per year (24). Looking at the 5-year stroke mortalities, the reduction has not yet been achieved.

The government passage of Sin Tax Reform Law in 2012, raised the excise taxes on tobacco products and effectively reduced tobacco use among smokers from 29.7% in 2009 to 23.8% in 2015 based on the results of the Global Adult Tobacco Survey (GATS) (25). In 2018, an excise tax on sugar sweetened beverages was launched (26) which could be one of the reasons for reduction of diabetes prevalence among age 20–79 from 9.7% in 2011 to 7.1% in 2021 (27).

In 2013, the DOH launched its Go4Health program, a nationwide healthy lifestyle movement that aims to inform and encourage Filipinos to practice a healthy lifestyle by making a personal commitment to avoid the four risk factors - unhealthy diet, physical inactivity, tobacco use and harmful use of alcohol and to promote and establish a sustainable environment for healthy lifestyle (28). Several community healthy lifestyle campaigns were also done by the different medical societies (29, 30).

### Stroke awareness campaigns

There is low stroke awareness in many regions of the country. In a community survey by Roxas et al. (31), only 34.4% were knowledgeable on stroke and respondents even misconstrued the disease with heart attack. The SSP held regular lay fora, television and radio campaigns on stroke awareness (32). The creation of stroke infomercial for health care facilities, movie houses and public places was also done (33–36). In addition, regular social media stroke campaigns on YouTube, Facebook and Instagram were also intensified (37–41).

### Stroke education

In 2015, the Stroke Society of the Philippines in collaboration with the WSO, embarked on a nationwide 5-year stroke training on Cardinal Principles of Stroke Treatment (CPOST). The aim of the training was to help organize stroke teams, develop stroke ready hospitals and acute stroke units (42). This was one of the first steps to organize and standardize stroke education and training among physicians and nurses in the different regions of the country. Thrombolysis simulation and case-based neuroimaging workshops were the highlights of the training (42).

In 2020, amidst the surging COVID pandemic, the Stroke Society of the Philippines continued to push its advocacy of improving stroke care throughout the Philippines by hybrid method. The project Bringing evidenced-based stroke treatment to Philippine hospitals (BEST-PH) continued to provide the needed training for health care facilities to become Acute Stroke Ready Hospitals (ASRH) (43). Focusing on the developing stroke policy frameworks and formation of interdisciplinary stroke teams, the project facilitated the education and training of health care workers on the safe stroke protocols, care algorithms and treatment guidelines. In addition, the BEST-Ph project was able to provide the groundwork for the formation of a Telestroke network in the different hospitals (43).

The ANGELS initiatives not only facilitated stroke training but provided standardized evidenced-based stroke protocols, checklists, thrombolysis mechanics, monitoring and after stroke care procedures (44). The SSP is in the process of developing the Philippine Clinical Practice Guidelines on acute stroke care and management.

### Setting up of acute stroke ready hospitals and certification

After the stroke education and training, there was an increase in the number of acute stroke ready hospitals (ASRH) and acute stroke units (ASU) in the different areas of the country. There are 53 ASRH and 47 ASU in the country (35). The stroke society is currently evaluating and beginning the process of certification so that the ASRH and ASU meet the highest standard of care.

Some hospitals with limited beds and where stroke units do not exist, patients with stroke are admitted to the general wards, staffed by a coordinated multidisciplinary team with training in stroke care.

### Improvement in thrombolysis rate

**Figure 3:**
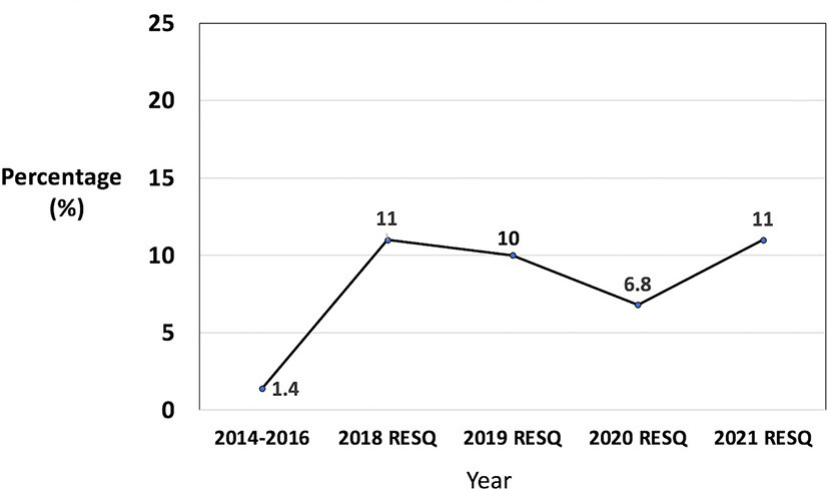
Thrombolysis rate (%), 2014–2021 (15, 45).

In 2014–2016, the thrombolysis rate (Figure 3) was low at 1.4% of all acute ischemic strokes. Through various stroke training and education in the different areas of the country by the stroke society, the thrombolysis rate increased to 11% in 2021 based on RES-Q database (45). The Res-Q database is participated by 27 ASRH hospitals but not all stroke cases are encoded as only a minimum of 30 representative cases per quarter are encoded. There was a slight decrease in the thrombolysis rate during the COVID pandemic in 2020. The PNA Stroke Database (45) is participated by 11 Neurology training institutions and encodes all stroke cases seen at the training hospitals. There is a higher thrombolysis rate in the public hospitals (Table 1) since the medicine rTPA is subsidized by the government. The initial cost of one vial of rTPA was 1,200 USD but in 2020, the cost was reduced to 600 USD through a government executive order regulating prices of drugs and medicines to improve access to health care (4).

**Table 1:** Average thrombolysis rates in different hospitals, PNA stroke database, 2021.

**% Thrombolysis**	**Government hospitals**	**Private hospitals**
PNA stroke database, 2021–2022 (4)	7.4% (range 4.4–16.9)	4.8% (range 0–10.1)

**Figure 4:**
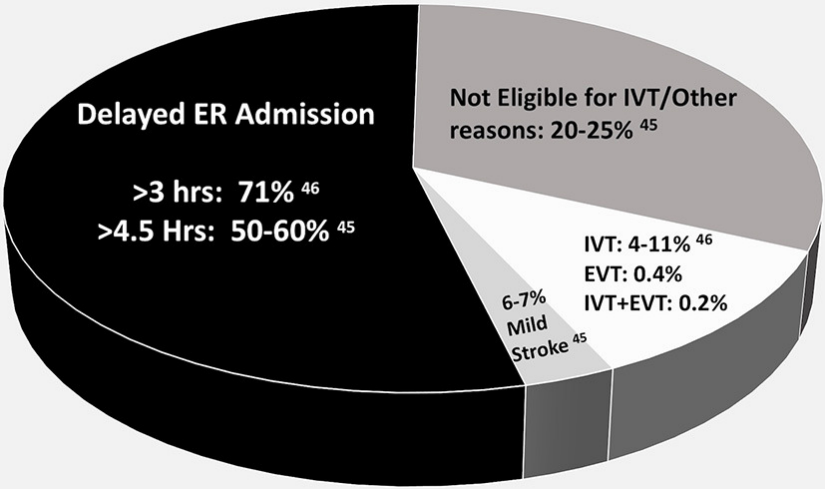
Patients thrombolyzed and not thrombolyzed among all acute ischemic strokes.

Of all the acute ischemic strokes patients, 50%−60% in the RES-Q database and 71% in the PNA Database arrive at the ER beyond 3 h from the time of stroke (Figure 4) (44, 45).

### Mechanical thrombectomy

The Philippines, together with Bangladesh, Mongolia, Nepal and Pakistan is one of the countries with limited EVT and IVT despite the high stroke burden (46). The high cost of EVT, lack of adequate stroke infrastructures, inadequate number of neuro-interventionists to perform the procedure, lack of unified territory -wide triage systems and inadequate awareness and education of both physicians and patients about this therapy are the identified impediments to thrombectomy (46). A retrospective study of a single center in Manila reported 31 thrombectomy cases among 924 ischemic stroke patients from 2018 to 2021 with discharge MRS 0–2 in 23%, 48% with MRS 3–5 and 29% mortality (47).

In the PNA Stroke Database, the annual thrombectomy rate of three comprehensive stroke centers in Manila is 0.4 and 0.2% for combined intravenous rTPA and endovascular therapy (4).

### Missed opportunities for improvement in stroke outcome

Figure 4, shows the percentage of delayed arrival to hospitals, with missed opportunities for IV thrombolysis. More than 50% of acute stroke patients have a delayed ER arrival time (44, 45). The reasons include poor stroke awareness, transportation problems and traffic, transfer from a primary hospital with minimal stroke care and incomplete facilities, overcrowding of public hospitals, lack of organized stroke network and lack of EMS (7).

### Stroke registry

An interoperable national stroke registry across public and private facilities is vital in improving stroke care. The Philippine Neurological Association One Database – Stroke (PNA1DB-Stroke; clinicaltrials.gov NCT04972058) started data collection on 1 June 2021 (45). This project is supported by the Philippine Neurological Association. This multi-center observational study includes all patients diagnosed with TIA or stroke, ≥18 years old, who are admitted in the country's 11 accredited adult neurology residency training institutions. Based on 2017–2019 census, ~10,000 cases each year may be included. As of 21 August 2022, 3,721 cases (mean age 58.1 ± 14.1 years, 42% women) have been registered, comprised of ~4% TIA, 58% ischemic stroke, 33% intracerebral hemorrhage, 5% subarachnoid hemorrhage and 0.1% cerebral venous thrombosis cases. Collective data spanning three years will be extracted, summarized and analyzed every year. Real-world data will be extremely valuable in identifying areas for improvement in stroke care by monitoring trends over the years and benchmarking against management guidelines. Findings are expected to guide public health policies and resource allocations. While situations in participating sites may not be fully representative of all hospitals in the country, the PNA1DB-Stroke may become a model that may be implemented in other designated stroke-ready hospitals.

Twenty seven (27) ASRH in the Philippine have joined the RES-Q database (45), a global registry to help physicians monitor and improve stroke care quality. Every hospital and country gets feedback for improvement. Acute stroke ready hospitals in the country have been given Angels award confirming compliance to the standard quality stroke care.

## Discussion

The National Stroke Policy will not resolve all issues as resources are needed. Emergency medical services (EMS) is a critical component and is needed to improve stroke outcome. In the Philippines, there is no EMS but limited number of ambulances to transport patients without protocols for field triage, standards of care or communication to receiving facilities. Legislations for the establishment of pre hospital emergency medical services including capacity building will facilitate time sensitive stroke treatment.

Technology can help bridge the gap in health care delivery in geographically isolated areas. Stroke education, training, stroke assessment, treatment and rehabilitation can be done online. Managing patients with acute ischemic stroke (AIS) using the guidance of telemedicine consultation is safe and reliable (48).

In rural and remote settings, mobile stroke units (MSU) may be adapted to local needs. In Thailand where the city has the worst traffic, the MSU decreased the alarm to needle time (49).

Telecommunication approaches between MSU and the stroke center can provide real time remote specialist advise. Stroke network with hub and spoke model can be adapted.

To address the limited number of neurologists, non-neurologists can be trained to do thrombolysis. In Thailand, to cope with the limited number of neurologists, they established stroke network project which assists the non-neurologists in the treatment of AIS with rtPA. Non-neurologists was able to thrombolyze AIS patients safely and effectively (50). Similar to Malaysia, non-neurologist hospitals may be able to provide thrombolysis service to AIS patients safely and effectively (51). In the Philippines, the neurologist can train the non-neurologists and form a stroke network, guiding the health care workers via telemedicine platform on acute stroke treatment. Although the thrombolysis rate has improved from 1.4 to 11%, this can still improve if the 50%−60% of acute ischemic stroke patients with delayed ER admission can be transported faster with EMS or organized stroke network.

The gap between LMIC and HIC is even more pronounced with endovascular treatment options for acute stroke. For the provision of mechanical thrombectomy, the center requires highly specialized staff, facilities and technical resources. In the Philippines, because of out of pocket expenses, admission to the comprehensive stroke centers are limited to those in the high economic strata. Similar to coronary artery stenting and bypass, the Philhealth insurance should provide funding for catastrophic illness like stroke with large vessel occlusion needing mechanical thrombectomy.

To address the limited number of rehabilitation facilities, home or community-based tele-rehabilitation may be used. In Nigeria, a video home based telerehabilitation was developed and found to be feasible, acceptable and useful among mild to moderate stroke survivors (52).

In low resource areas, greater resources should be allocated for stroke prevention. Primary stroke prevention reduces prevalence and incidence preventing need for a tertiary care, saving enormous expenses. This requires both community-based and government-based approaches to prevention. It is important to recognize the gravity of stroke pandemic so that urgent measures and strategies are done for prevention and treatment.

## Conclusion

Strategies to address stroke care gaps included community awareness, training of neurologists and nurses on acute stroke care, standardization of stroke protocols, setting up of ASRH and ASU and stroke registries. However, there is still a need to increase community stroke awareness, upgrading in Philippine health insurance, development of adequate stroke infrastructures and network which will greatly improve stroke outcomes.

### Limitations

The data were taken from limited number of published studies on stroke in the Philippines. Data from stroke registries may not reflect the real situation in the country as majority of the ASRH participating in the stroke database are in the urban areas where health care is better.

## Author contributions

MC, JN, RG, and AB took the lead in preparing the draft manuscript for publication. All authors participated in the data gathering and provided input in developing the manuscript and approved the final version submitted.

## Conflict of interest

The authors declare that the research was conducted in the absence of any commercial or financial relationships that could be construed as a potential conflict of interest.

## Publisher's note

All claims expressed in this article are solely those of the authors and do not necessarily represent those of their affiliated organizations, or those of the publisher, the editors and the reviewers. Any product that may be evaluated in this article, or claim that may be made by its manufacturer, is not guaranteed or endorsed by the publisher.
